# Efficacy of azithromycin sequential therapy combined with montelukast in the treatment of pediatric mycoplasma pneumonia: A meta-analysis

**DOI:** 10.1097/MD.0000000000050050

**Published:** 2026-07-31

**Authors:** Mingming Wu, Kan Chen, Haihong Yu

**Affiliations:** a Pediatrics Department, Jiangcun Subdistrict Community Health Service Center, Hangzhou, Zhejiang Province, China; b General Practice Department, Jiangcun Subdistrict Community Health Service Center, Hangzhou, Zhejiang Province, China; c Department of Science and Education, Jiangcun Subdistrict Community Health Service Center, Hangzhou, Zhejiang Province, China.

**Keywords:** azithromycin, children, meta-analysis, montelukast sodium, Mycoplasma pneumoniae pneumonia

## Abstract

**Background:**

Mycoplasma pneumoniae pneumonia (MPP) stands as a predominant cause of community-acquired respiratory infections in children. With the increasing prevalence of macrolide-resistant Mycoplasma pneumoniae strains, the therapeutic efficacy of azithromycin monotherapy has become a growing concern in clinical practice. Montelukast sodium, a selective leukotriene receptor antagonist, has emerged as a promising adjunctive agent, as it can effectively alleviate airway inflammation and reduce airway hyperresponsiveness. This meta-analysis was designed to systematically evaluate the efficacy and safety of azithromycin sequential therapy combined with montelukast in the management of pediatric MPP.

**Methods:**

A comprehensive literature search was conducted across PubMed, Embase, Web of Science Core Collection, Wanfang Data, Variant Impact Predictor database, and Chinese National Knowledge Infrastructure from the establishment of each database up to November 2025. Eligible studies were randomized controlled trials (RCTs) that compared azithromycin sequential monotherapy with azithromycin sequential therapy plus montelukast in pediatric patients diagnosed with MPP. Primary outcome measures included overall clinical efficacy, resolution time of key clinical symptoms (cough, wheezing, pulmonary rales, and fever), and the incidence of adverse events.

**Results:**

Thirty-six RCTs involving 3450 pediatric patients (1726 in the combination group and 1724 in the azithromycin group) met the inclusion criteria. Compared with azithromycin sequential therapy, combination therapy significantly improved overall clinical efficacy (odds ratio = 4.75, 95% confidence interval [CI]: 3.70–6.10). The addition of montelukast also shortened the resolution time of cough (mean difference [MD] = −3.63, 95% CI: −4.27 to −2.99), wheezing (MD = −1.48, 95% CI: −1.82 to −1.14), pulmonary rales (MD = −2.06, 95% CI: −2.54 to −1.57), and fever (MD = −1.43, 95% CI: −1.89 to −0.96). No significant difference in adverse event rates was observed between groups.

**Conclusions:**

Azithromycin sequential therapy combined with montelukast may provide additional therapeutic benefits for the treatment of pediatric MPP, characterized by higher overall clinical efficacy and more rapid resolution of clinical symptoms, without a statistically significant increase in short-term adverse events. Despite the encouraging findings, the included studies were predominantly single-center trials with moderate methodological quality. Therefore, well-designed, large-scale, multicenter RCTs are urgently needed to validate these results and provide more reliable evidence for guiding clinical decision-making.

## 1. Introduction

Mycoplasma pneumoniae pneumonia (MPP) is one of the most common community-acquired respiratory infections in children, accounting for approximately 10% to 40% of pediatric pneumonia cases worldwide.^[1]^ Although macrolides – particularly azithromycin – remain the 1st-line therapy due to their ability to inhibit mycoplasmal protein synthesis, the widespread use of macrolides in recent years has led to a notable rise in macrolide-resistant strains, resulting in suboptimal treatment responses among many pediatric patients.^[2]^ Children with MPP often present with persistent dry cough, wheezing, and airway hyperresponsiveness, which may last for weeks or months and substantially impair quality of life. Consequently, adjunctive anti-inflammatory and bronchodilator interventions have gained increasing clinical attention.^[3]^ Montelukast, a leukotriene receptor antagonist, exerts targeted anti-inflammatory effects by blocking cysteinyl leukotriene-mediated airway edema, mucus secretion, and bronchoconstriction, thereby improving airway reactivity and symptom resolution.^[4]^ Emerging clinical studies published in recent years consistently suggest that the combination of azithromycin and montelukast provides superior benefits compared with azithromycin monotherapy, including shorter cough and wheezing duration, improved pulmonary function and radiological findings, and better modulation of inflammatory and immune responses.^[5]^ To systematically evaluate the current evidence, this meta-analysis investigates the therapeutic efficacy and safety of azithromycin sequential therapy plus montelukast in children with MPP, with the aim of providing robust, evidence-based support for optimized clinical management.

## 2. Materials and methods

### 2.1. Data sources and search strategy

This study was approved by the Ethics Committee of Jiangcun Subdistrict Community Health Service Center of Xihu District. A comprehensive literature search was performed in PubMed, Embase, Wanfang, Web of Science Core Collection, Variant Impact Predictor database, and Chinese National Knowledge Infrastructure database from their inception to November 2025 to identify randomized controlled trials (RCTs) evaluating montelukast sodium combined with azithromycin in pediatric MPP.

A combination of subject terms and free-text terms was used.Search terms included “children,” “Mycoplasma pneumoniae,” “azithromycin,” and “montelukast sodium.” Search strategies were refined through several pre-searches by 2 independent reviewers. Reference lists of included articles were manually screened to identify additional eligible studies. This review was not prospectively registered in PROSPERO; the absence of registration is acknowledged as a limitation.

### 2.2. Inclusion and exclusion criteria

1. Study type: RCTs only, published in Chinese or English.
2. Population: Children (0–14 years) diagnosed with MPP according to authoritative pediatric guidelines, such as the Expert Consensus on the Diagnosis and Treatment of MPP (or other relevant guidelines).
3. Intervention: The control group received sequential therapy with azithromycin, while the experimental group was administered montelukast in combination with the same azithromycin sequential therapy as the control group.
4. Comparator: The control group received azithromycin sequential therapy.
5. Outcomes: Primary outcome: Overall clinical effective rate. Secondary outcomes: Time to resolution of cough, wheezing, fever, and pulmonary rales; incidence of adverse reactions.
6. Exclusion criteria included: Non-RCTs, duplicated publications, incomplete or unverifiable data, unclear diagnostic or efficacy criteria, and reviews or case reports, as well as studies in which additional antibiotics or glucocorticoids were used unequally between groups or could not be verified. Routine supportive care such as antipyretics, nebulization, oxygen therapy, or bronchodilators was accepted only when reported as comparable between groups.

### 2.3. Literature screening, data extraction, and quality assessment

1. Two reviewers independently screened titles, abstracts, and full texts according to predetermined criteria. Disagreements were resolved through discussion or adjudication by a 3rd reviewer. Extracted information included the 1st author and publication year; sample size and baseline characteristics; intervention details; outcome measures; and methodological characteristics of each study. The risk of bias of included RCTs was assessed using the Cochrane Risk of Bias Tool 2.0, evaluating^[6]^:
2. Random sequence generation, allocation concealment, blinding of participants and personnel, blinding of outcome assessors, completeness of outcome data, selective reporting, and other potential biases. The “other bias” domain was rated as high risk when important baseline comparability, co-intervention control, treatment-course consistency, or outcome-measurement details were insufficiently reported and could plausibly influence the pooled effect estimate.

### 2.4. Outcome definitions

The overall clinical effective rate was calculated as (markedly effective + effective)/total cases. In most trials, “markedly effective” referred to complete or near-complete resolution of symptoms and radiographic lesions, whereas “effective” referred to substantial symptom improvement and partial radiographic improvement. Because the original trials did not always use fully identical clinical-efficacy criteria, this outcome was interpreted cautiously. Symptom-resolution time was defined as the number of days from treatment initiation to the disappearance of cough, wheezing, fever, or pulmonary rales. Adverse events included gastrointestinal discomfort, rash, abnormal liver function, and other events reported by the original studies.

### 2.5. Statistical analysis

Meta-analysis was conducted using RevMan version 5.4.1 (The Cochrane Collaboration) dichotomous outcomes, including overall clinical effective rate and adverse events, were expressed as odds ratios (ORs) with 95% confidence intervals (CIs). Continuous outcomes, including symptom-resolution time, were expressed as mean differences (MD) with 95% CI. Heterogeneity was evaluated using the χ^2^ test and *I*^2^ statistic. Fixed-effects models were used when heterogeneity was low (*I*^2^ < 50%, *P* > .1). Random-effects models were applied when substantial heterogeneity existed (*I*^2^ ≥ 50% or *P* < .1).

## 3. Results

### 3.1. Study selection and characteristics of included studies

**Figure 1.**
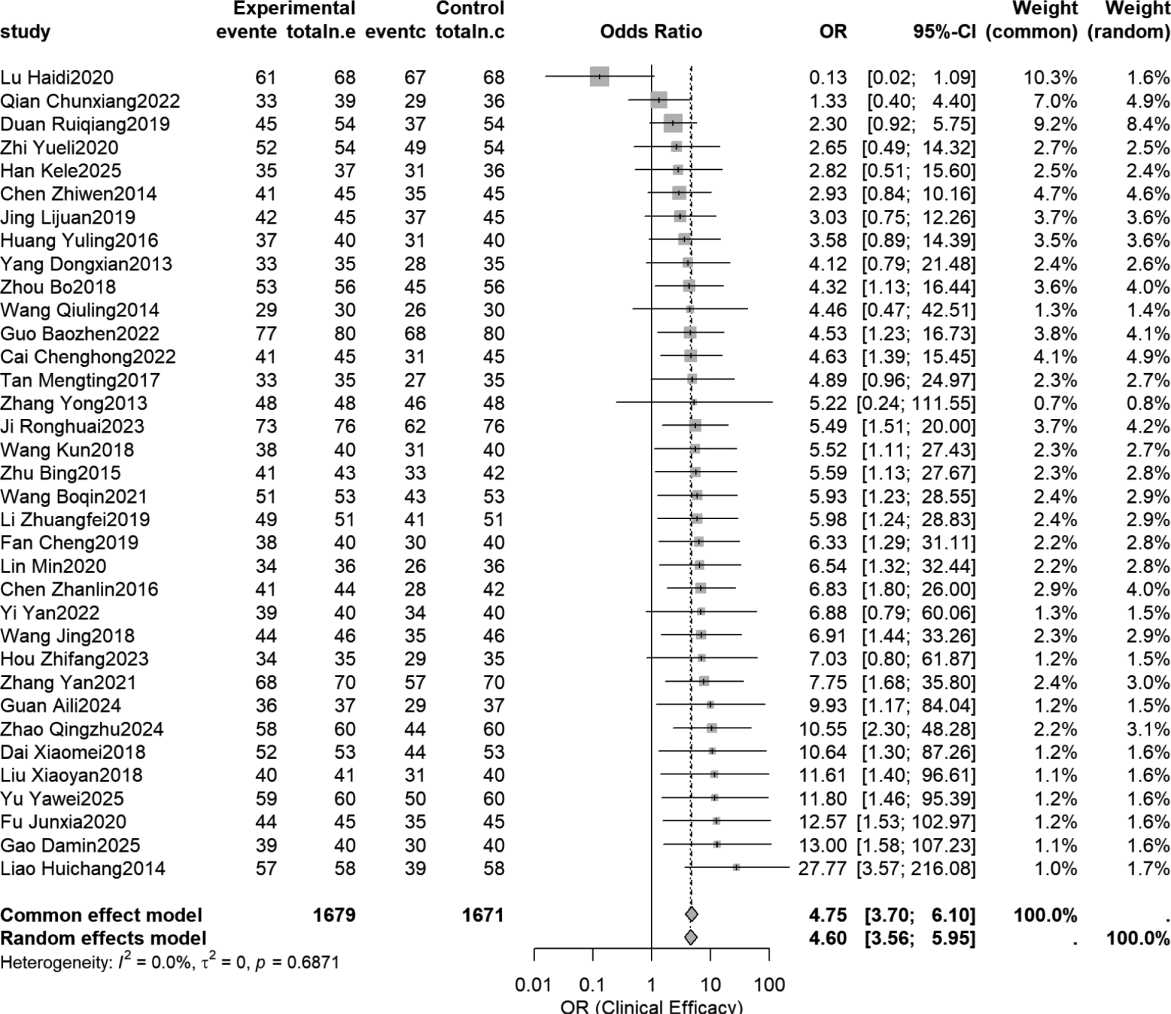
Forest map of clinical efficiency meta-analysis. CI = confidence interval, OR = odds ratio.

Note: The specific databases retrieved and the number of identified documents are as follows: PubMed (n = 2), Embase (n = 0), Wanfang (n = 88), Web of Science Core Collection (n = 1), Variant Impact Predictor (n = 76) , and Chinese National Knowledge Infrastructure (n = 207).

The initial database search identified a total of 374 records. After removing duplicates and screening titles and abstracts, 131 articles were retained for full-text evaluation. Of these, 95 studies were excluded for reasons such as non-randomized design, inconsistent interventions, or insufficient outcome reporting. Ultimately, 36 randomized controlled trials^[7–42]^ published between 2013 and 2025, involving a total of 3450 pediatric patients, met the inclusion criteria and were included in the meta-analysis. Among them, 1726 children received azithromycin combined with montelukast, while 1724 received azithromycin sequential therapy. The Preferred Reporting Items for Systematic Reviews and Meta-Analyses flow diagram of the screening process is shown in Figure 1. Key study characteristics are summarized in Table 1 reporting items for systematic reviews and meta-analyses.

**Table 1:** The basic features of studies included in the meta-analysis.

Included study	n/cases		Treatment strategy		Treatment time (d)	Evaluation index
	T	C	T	C		
Yang Dongxian 2013^[7]^	35	35	C + M	A (ivgtt + po)	7	①⑥
Zhang Yong 2013^[8]^	48	48	C + M	A (ivgtt + po)	23	①②④⑤
Chen Zhiwen 2014^[9]^	45	45	C + M	A (ivgtt + po)	23	①②⑤⑥
Liao Huichang 2014^[10]^	58	58	C + M	A (ivgtt + po)	21	①②⑥
Wang Qiuling 2014^[11]^	30	30	C + M	A (ivgtt + po)	14	①②④⑤
Zhu Bing 2015^[12]^	43	42	C + M	A (ivgtt + po)	28	①②③④⑤
Chen Zhanlin 2016^[13]^	44	42	C + M	A (ivgtt + po)	21	①②③④⑤⑥
Huang Yuling 2016^[14]^	40	40	C + M	A (ivgtt + po)	21	①②④⑤
Tan Mengting 2017^[15]^	35	35	C + M	A (ivgtt + po)	21	①⑦
Dai Xiaomei 2018^[16]^	53	53	C + M	A (ivgtt + po)	21	①⑦
Liu Xiaoyan 2018^[17]^	41	40	C + M	A (ivgtt + po)	30	①②④⑤⑦
Wang Jing 2018^[18]^	46	46	C + M	A (ivgtt + po)	28	①⑦
Wang Kun 2018^[19]^	40	40	C + M	A (ivgtt + po)	28	①⑦
Zhou Bo 2018^[20]^	56	56	C + M	A (ivgtt + po)	28	①⑦
Duan Ruiqiang 2019^[21]^	54	54	C + M	A (ivgtt + po)	21	①
Fan Cheng 2019^[22]^	40	40	C + M	A (ivgtt + po)	21	①②③④⑤⑥⑦
Jing Lijuan 2019^[23]^	45	45	C + M	A (ivgtt + po)	28	①②③④⑤⑦
Li Zhuangfei 2019^[24]^	51	51	C + M	A (ivgtt + po)	21	①⑦
Fu Junxia 2020^[25]^	45	45	C + M	A (ivgtt + po)	7	①⑦
Zhi Yueli 2020^[26]^	54	54	C + M	A (ivgtt + po)	7	①④⑤⑦
Liu Ying 2020^[27]^	50	50	C + M	A (ivgtt + po)	21	②③④⑤⑥
Lu Haidi 2020^[28]^	68	68	C + M	A (ivgtt + po)	10	①②③④⑤
Lin Min 2020^[29]^	36	36	C + M	A (ivgtt + po)	14	①②③④⑤⑥
Zhang Yan 2021^[30]^	70	70	C + M	A (ivgtt + po)	14	①⑦
Wang Boqin 2021^[31]^	53	53	C + M	A (ivgtt + po)	21	①⑥⑦
Yi Yan 2021^[32]^	40	40	C + M	A (ivgtt + po)	14	①②④⑤⑥
Guo Baozhen 2022^[33]^	80	80	C + M	A (ivgtt + po)	14	①②③④⑤⑥⑦
Cai Chenghong 2022^[34]^	45	45	C + M	A (ivgtt + po)	10	①⑦
Qian Chunxiang 2022^[35]^	36	39	C + M	A (ivgtt + po)	21	①②④⑤⑥
Hou Zhifang 2023^[36]^	35	35	C + M	A (ivgtt + po)	28	①②③④⑤⑦
Ji Ronghuai 2023^[37]^	76	76	C + M	A (ivgtt + po)	14	①②③④⑤⑦
Zhao Qingzhu 2024^[38]^	60	60	C + M	A (ivgtt + po)	21	①②③④⑤⑥
Guan Aili 2024^[39]^	37	37	C + M	A (ivgtt + po)	21	①②③④⑤⑥
Yu Yawei 2025^[40]^	60	60	C + M	A (ivgtt + po)	21	①②③④⑤⑦
Gao Damin 2025^[41]^	40	40	C + M	A (ivgtt + po)	21	①②④⑤⑥⑦
Han Kele 2025^[42]^	37	36	C + M	A (ivgtt + po)	14	①②③④⑤⑥

Evaluation indicators included: ① clinical total effective rate; ② cough disappearance time; ③ wheezing disappearance time; ④ fever disappearance time; ⑤ pulmonary rales disappearance time; ⑥ adverse reactions; ⑦ other indicators such as pulmonary function.

A = azithromycin, C = control group, ivgtt = intravenous drip, M = montelukast sodium, po = oral administration, T = treatment group.

Treatment strategies: group T received montelukast sodium combined with azithromycin, while group C received Azithromycin alone; the administration routes included intravenous drip and oral administration.

### 3.2. Methodological quality assessment

Except for Huang Yuling 2016,which did not report randomization, all included trials stated the use of random allocation. However, most studies did not provide sufficient information on allocation concealment or blinding. Only Yu Yawei 2025 provided clear details on allocation concealment and participant blinding. No trial was judged to have high risk because of incomplete outcome data or selective reporting.Zhu Bing 2015, Chen Zhanlin 2016, Liu Xiaoyan 2018, Qian Chunxiang 2022 and Han Kele 2025 were rated as high risk for other bias because of insufficient reporting of baseline comparability, co-intervention control, treatment-course consistency, or outcome-measurement details. Overall, the methodological quality of the included studies was moderate. The risk-of-bias assessment is presented in Table 2.

**Table 2:** Risk of bias assessment for RCTs.

Study	Random sequence	Assignment hiding	Participant and practitioner blindness	Outcome assessment blinding	Incomplete ending data	Selective reporting	Other biases
Yang Dongxian 2013^[7]^	Low	Unclear	Unclear	Unclear	Low	Low	Low
Zhang Yong 2013^[8]^	Low	Unclear	Unclear	Unclear	Low	Low	Low
Chen Zhiwen 2014^[9]^	Low	Unclear	Unclear	Unclear	Low	Low	Low
Liao Huichang 2014^[10]^	Low	Unclear	Unclear	Unclear	Low	Low	Low
Wang Qiuling 2014^[11]^	Low	Unclear	Unclear	Unclear	Low	Low	Low
Zhu Bing 2015^[12]^	Low	Unclear	Unclear	Unclear	Low	Low	High
Chen Zhanlin 2016^[13]^	Low	Unclear	Unclear	Unclear	Low	Low	High
Huang Yuling 2016^[14]^	High	Unclear	Unclear	Unclear	Low	Low	Low
Tan Mengting 2017^[15]^	Low	Unclear	Unclear	Unclear	Low	Low	Low
Dai Xiaomei 2018^[16]^	Low	Unclear	Unclear	Unclear	Low	Low	Low
Liu Xiaoyan 2018^[17]^	Low	Unclear	Unclear	Unclear	Low	Low	High
Wang Jing 2018^[18]^	Low	Unclear	Unclear	Unclear	Low	Low	Low
Wang Kun 2018^[19]^	Low	Unclear	Unclear	Unclear	Low	Low	Low
Zhou Bo 2018^[20]^	Low	Unclear	Unclear	Unclear	Low	Low	Low
Duan Ruiqiang 2019^[21]^	Low	Unclear	Unclear	Unclear	Low	Low	Low
Fan Cheng 2019^[22]^	Low	Unclear	Unclear	Unclear	Low	Low	Low
Jing Lijuan 2019^[23]^	Low	Unclear	Unclear	Unclear	Low	Low	Low
Li Zhuangfei 2019^[24]^	Low	Unclear	Unclear	Unclear	Low	Low	Low
Fu Junxia 2020^[25]^	Low	Unclear	Unclear	Unclear	Low	Low	Low
Zhi Yueli 2020^[26]^	Low	Unclear	Unclear	Unclear	Low	Low	Low
Liu Ying 2020^[27]^	Low	Unclear	Unclear	Unclear	Low	Low	Low
Lu Haidi 2020^[28]^	Low	Unclear	Unclear	Unclear	Low	Low	Low
Lin Min 2020^[29]^	Low	Unclear	Unclear	Unclear	Low	Low	Low
Zhang Yan 2021^[30]^	Low	Unclear	Unclear	Unclear	Low	Low	Low
Wang Boqin 2021^[31]^	Low	Unclear	Unclear	Unclear	Low	Low	Low
Yi Yan 2021^[32]^	Low	Unclear	Unclear	Unclear	Low	Low	Low
Guo Baozhen 2022^[33]^	Low	Unclear	Unclear	Unclear	Low	Low	Low
Cai Chenghong 2022^[34]^	Low	Unclear	Unclear	Unclear	Low	Low	Low
Qian Chunxiang 2022^[35]^	Low	Unclear	Unclear	Unclear	Low	Low	High
Hou Zhifang 2023^[36]^	Low	Unclear	Unclear	Unclear	Low	Low	Low
Ji Ronghuai 2023^[37]^	Low	Unclear	Unclear	Unclear	Low	Low	Low
Zhao Qingzhu 2024^[38]^	Low	Unclear	Unclear	Unclear	Low	Low	Low
Guan Aili 2024^[39]^	Low	Unclear	Unclear	Unclear	Low	Low	Low
Yu Yawei 2025^[40]^	Low	Low	Low	Unclear	Low	Low	Low
Gao Damin 2025^[41]^	Low	Unclear	Unclear	Unclear	Low	Low	Low
Han Kele 2025^[42]^	Low	Unclear	Unclear	Unclear	Low	Low	High

RCT = randomized controlled trial.

### 3.3. Clinical efficacy

Thirty-five studies reported the overall clinical effective rate. No significant heterogeneity was observed among the included studies; *I*^2^ = 0.0 therefore, a fixed-effects model was used for the primary pooled analysis. The pooled results showed that azithromycin sequential therapy combined with montelukast significantly improved the overall clinical effective rate compared with azithromycin sequential therapy alone (OR = 4.75, 95% CI: 3.70–6.10, *P* < .001, Fig. 1). The result remained consistent when a random-effects model was applied (OR = 4.60, 95% CI: 3.56–5.95), indicating that the pooled estimate was robust.

### 3.4. Symptom relief outcomes

#### 3.4.1. Time to cough resolution

**Figure 2.**
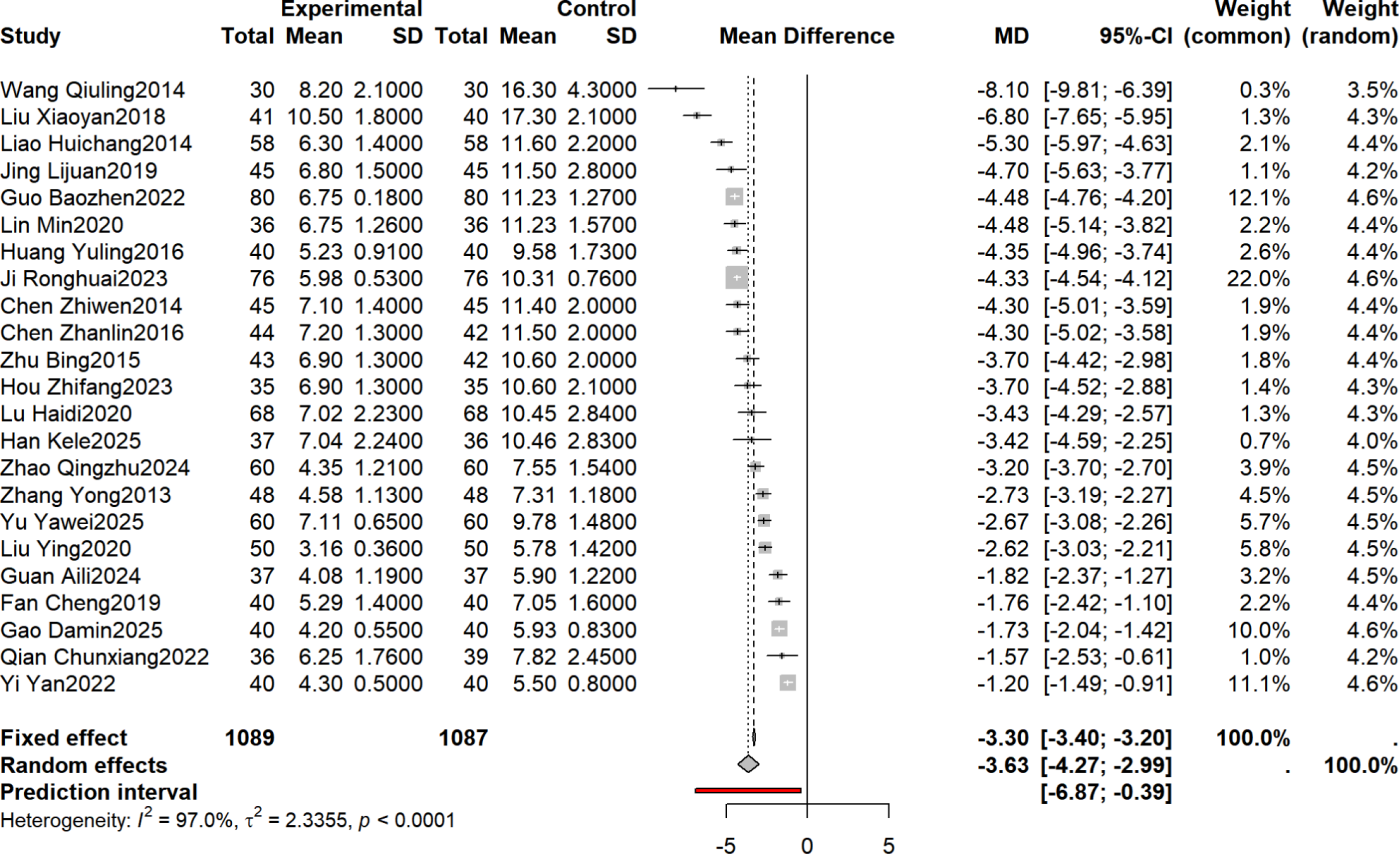
Forest map of cough resolution time. CI = confidence interval, MD = mean difference, SD = standard deviation.

Twenty-three studies reported cough resolution time. Substantial heterogeneity was observed among the included studies *I*^2^ = 97%; therefore, a random-effects model was used. The pooled results showed that azithromycin sequential therapy combined with montelukast significantly shortened cough resolution time compared with azithromycin sequential therapy alone (MD = −3.63 days, 95% CI: −4.27 to −2.99, *P* < .0001, Fig. 2).

#### 3.4.2. Time to wheeze resolution

**Figure 3.**
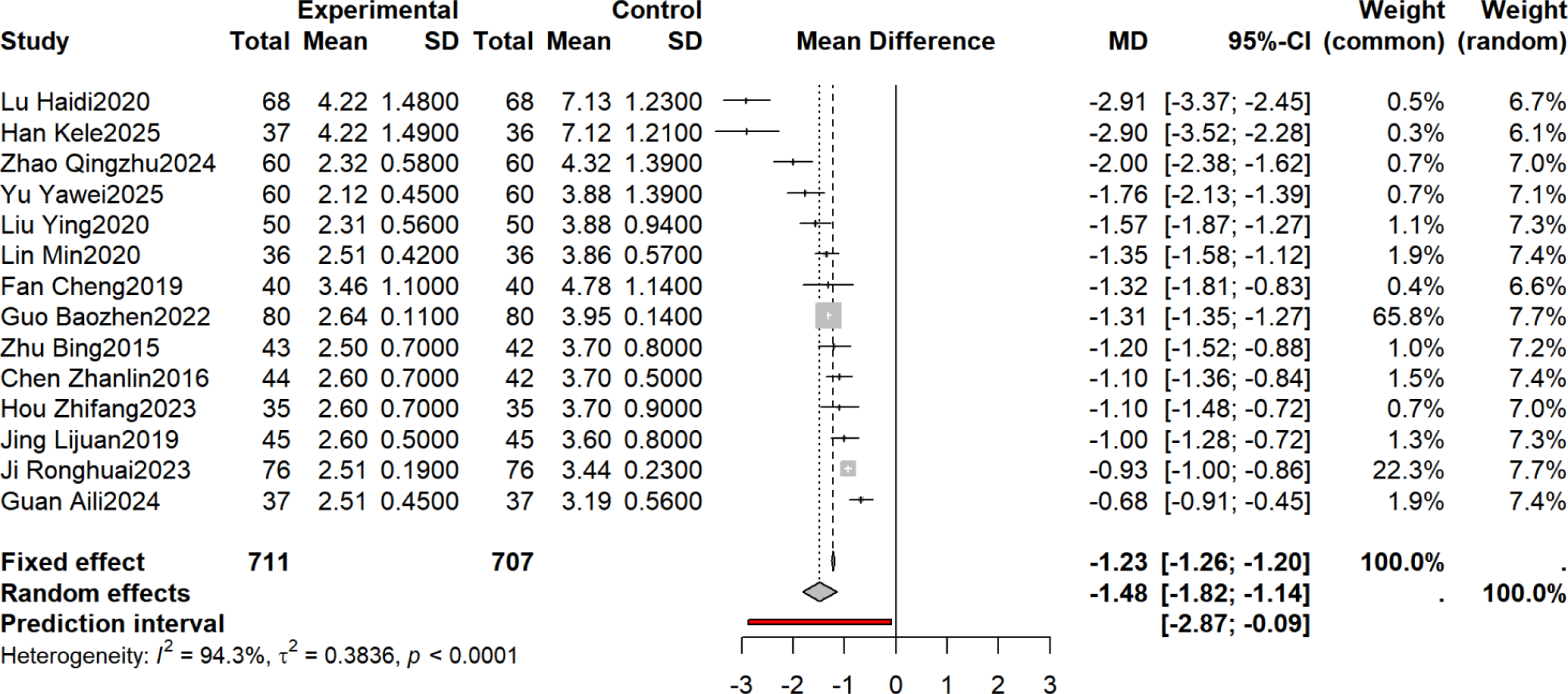
Forest map of time to disappearance of wheezing. CI = confidence interval, MD = mean difference, SD = standard deviation.

Fourteen studies involving 1418 children reported the time to wheezing resolution. Substantial heterogeneity was observed among the included studies; *I*^2^ = 94.3 therefore, a random-effects model was applied. The pooled results showed that azithromycin sequential therapy combined with montelukast significantly shortened the time to wheezing resolution compared with azithromycin sequential therapy alone (MD = −1.48 days, 95% CI: −1.82 to −1.14, *P* < .0001, Fig. 3).

#### 3.4.3. Time to disappearance of pulmonary rales

**Figure 4.**
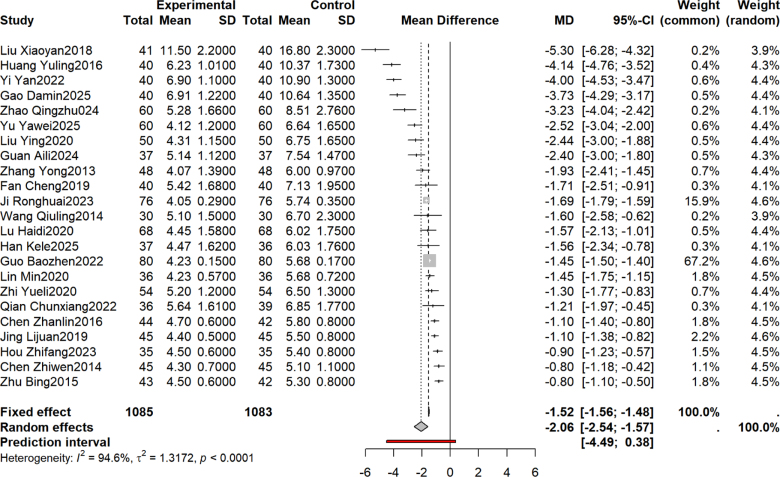
Forest map of time to disappearance of lung rales. CI = confidence interval, MD = mean difference, SD = standard deviation.

Twenty-three studies reported the duration of pulmonary rales. Substantial heterogeneity was detected among the included studies; *I*^2^ = 94.6 therefore, a random-effects model was used. The pooled analysis indicated that azithromycin sequential therapy combined with montelukast significantly reduced the duration of pulmonary rales compared with azithromycin sequential therapy alone (MD = −2.06 days, 95% CI: −2.54 to −1.57, *P* < .0001, Fig. 4).

#### 3.4.4. Time to fever resolution

**Figure 5.**
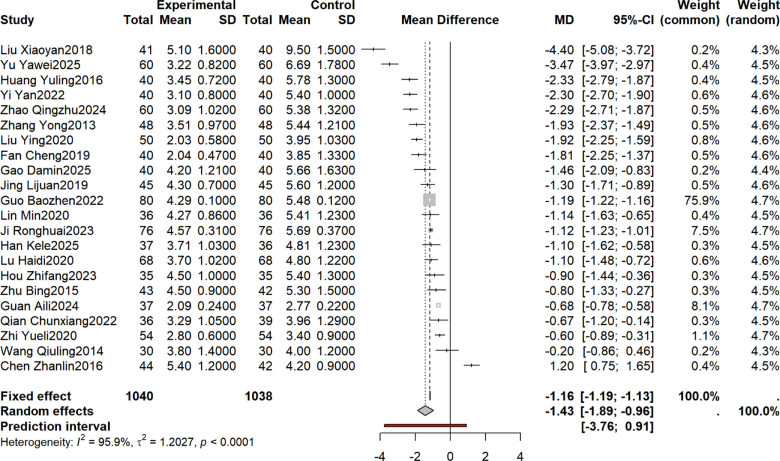
Forest map of time to fever resolution. CI = confidence interval, MD = mean difference, SD = standard deviation.

Twenty-two studies reported the time to fever resolution. Substantial heterogeneity was observed among the included studies; *I*^2^ = 95.9 therefore, a random-effects model was applied. The pooled results showed that azithromycin sequential therapy combined with montelukast significantly shortened fever resolution time compared with azithromycin sequential therapy alone (MD = −1.43 days, 95% CI: −1.89 to −0.96, *P* < .0001, Fig. 5).

### 3.5. Adverse events

**Figure 6.**
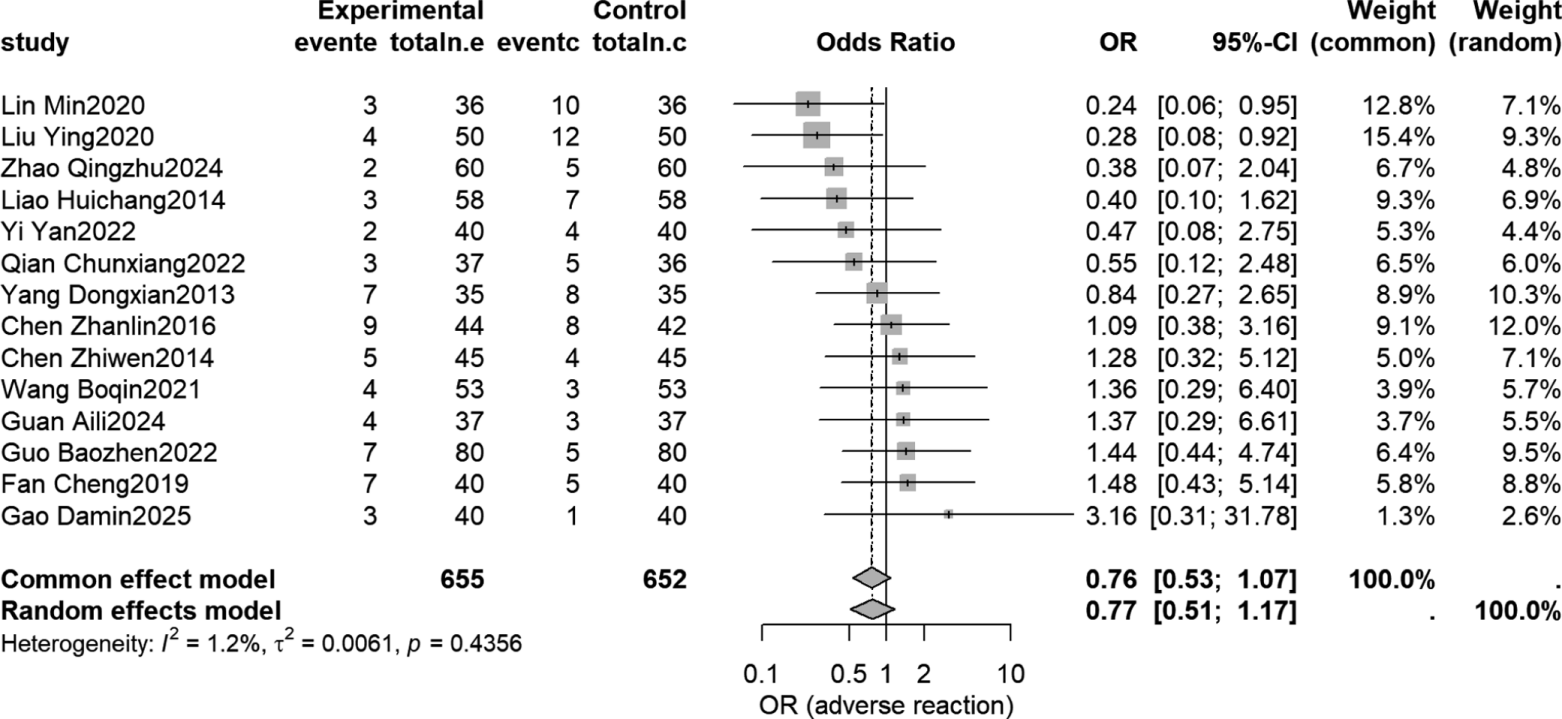
Forest map of meta-analysis of adverse reaction rate. CI = confidence interval, OR = odds ratio.

Fourteen studies reported adverse events. There was no significant heterogeneity among the included studies (*I*^2^ = 1.2%, *P* = .4356); therefore, a fixed-effect model was used. The pooled estimate did not show a statistically significant difference in adverse event incidence between azithromycin sequential therapy combined with montelukast and azithromycin sequential therapy alone (OR = 0.76, 95% CI: 0.53–1.07, *P* = .12, Fig. 6). Although the point estimate numerically favored the combination group, the confidence interval crossed 1.0; therefore, the difference was interpreted as not statistically significant. The most commonly reported adverse events were gastrointestinal discomfort and rash. However, because the original trials generally had brief follow-up and relied on short-term adverse-event reporting, delayed, rare, or neuropsychiatric events could not be adequately evaluated.

### 3.6. Heterogeneity, sensitivity analysis, publication bias, and certainty of evidence

**Figure 7.**
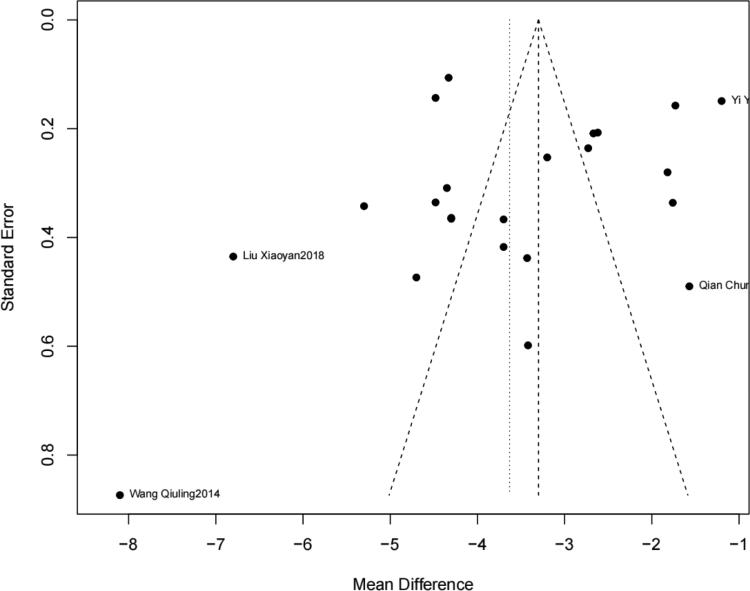
Funnel plot for cough resolution time.

Substantial heterogeneity was observed for symptom-resolution outcomes. Potential sources included differences in age distribution, baseline pneumonia severity, azithromycin route and duration, montelukast dose, supportive care, and outcome-measurement criteria. Quantitative meta-regression was not feasible because these variables were not reported consistently across trials. Sensitivity analysis using an alternative random-effects model for the primary efficacy outcome yielded a consistent estimate (OR = 4.60, 95% CI: 3.56–5.95), supporting the robustness of the main finding. Publication bias for cough resolution time was assessed using a funnel plot and Egger regression test. Egger regression test did not show significant funnel plot asymmetry or small-study effects (*t* = −0.66, df = 21, *P* = .5145; intercept = −1.7173, standard error = 2.5899). However, because substantial residual heterogeneity was present, publication bias could not be completely excluded, and the funnel plot should be interpreted cautiously (Fig. 7). According to Grading of Recommendations Assessment (GRADE), the certainty of evidence was moderate for overall clinical efficacy, low for symptom-resolution outcomes, and low for short-term adverse events (Table 3) *I*^2^ = 97.0_._

**Table 3:** GRADE summary of evidence for primary outcomes.

Outcome	Studies	Pooled estimate	Certainty of evidence	Main reasons for rating
Overall clinical effective rate	35 RCTs	OR = 4.75, 95% CI: 3.70–6.10	Moderate	Downgraded for risk of bias; the effect estimate was consistent with negligible heterogeneity.
Cough resolution time	23 RCTs	MD = −3.63 d, 95% CI: −4.27 to −2.99	Low	Downgraded for risk of bias and very high heterogeneity.
Wheezing resolution time	14 RCTs	MD = −1.48 d, 95% CI: −1.82 to −1.14	Low	Downgraded for risk of bias and substantial heterogeneity.
Pulmonary rales disappearance time	23 RCTs	MD = −2.06 d, 95% CI: −2.54 to −1.57	Low	Downgraded for risk of bias and substantial heterogeneity.
Fever resolution time	22 RCTs	MD = −1.43 d, 95% CI: −1.89 to −0.96	Low	Downgraded for risk of bias and very high heterogeneity.
Adverse events	14 RCTs	OR = 0.76, 95% CI: 0.53–1.07	Low	Downgraded for risk of bias, imprecision, short follow-up, and possible underreporting.

CI = confidence interval, GRADE = Grading of Recommendations Assessment, MD = mean difference, OR = odds ratio, RCT = randomized controlled trial.

Heterogeneity, sensitivity analysis, publication bias, and development and evaluation (GRADE) certainty.

GRADE summary of evidence for primary outcomes.

## 4. Discussion

This meta-analysis demonstrated that adding montelukast to sequential azithromycin therapy confers additional clinical benefits in children with MPP. Compared with azithromycin alone, the combination regimen significantly improved overall clinical efficacy and accelerated the resolution of cough, wheezing, pulmonary rales, and fever. These findings suggest that supplementing macrolide-based sequential therapy with anti-inflammatory modulation may better address the multifactorial pathophysiology of MPP.

Given that azithromycin primarily targets pathogen replication, its therapeutic effect may be insufficient when airway inflammation, mucus hypersecretion, bronchial hyperresponsiveness, and immune-mediated injury persist after infection. The additional benefit observed in the combination group may therefore be partly explained by the pharmacological properties of montelukast.^[43]^ As a selective cysteinyl leukotriene receptor 1 antagonist, montelukast can attenuate leukotriene-driven airway inflammation and airway hyperresponsiveness. This mechanism is consistent with the observed improvements in cough and wheezing resolution, because these symptoms are closely associated with airway inflammatory mediator release and bronchial hyperreactivity.^[44]^ In addition, montelukast co-administration may be associated with greater reductions in C-reactive protein, tumor necrosis factor-alpha, interleukin-6, and immunoglobulin levels compared with macrolide monotherapy, suggesting broader modulation of both innate and adaptive immune responses.^[45]^

From a pharmacokinetic perspective, montelukast is mainly metabolized by hepatic CYP2C8, with minor contributions from CYP3A4 and CYP2C9, whereas azithromycin has relatively weak inhibitory activity on CYP3A4 compared with erythromycin or clarithromycin. Therefore, a major pharmacokinetic interaction between azithromycin and montelukast is unlikely. Nevertheless, because children with MPP may receive other concomitant medications, potential drug interactions and hepatic function should still be considered in clinical practice. Some clinical studies have also reported reductions in eosinophil counts, platelet levels, myocardial enzyme markers, and hepatic injury indicators after adjunctive montelukast therapy, suggesting potential systemic anti-inflammatory effects.^[46]^

In China, younger children – especially those under 4 years – frequently experience mixed respiratory infections involving Mycoplasma pneumoniae together with respiratory syncytial virus, Chlamydia trachomatis, or Streptococcus pneumoniae.^[47]^ Such coinfections can intensify airway inflammation and prolong recovery. The enhanced therapeutic effect observed with azithromycin plus montelukast may therefore be particularly relevant in these clinically complex cases.

## 5. Limitations

This study has several limitations. First, the included trials were mostly single-center studies with moderate or low methodological quality; allocation concealment and blinding were unclear in most trials, which may introduce performance and detection bias, especially for subjective outcomes such as symptom disappearance time. Second, substantial heterogeneity was present in cough, wheezing, fever, and pulmonary-rale resolution outcomes, and the original trials did not consistently report age strata, baseline severity, dosing regimens, route and duration of azithromycin, or supportive treatments, preventing robust subgroup analysis or meta-regression. Third, long-term follow-up data were limited, so recurrence, persistent cough, postinfectious airway hyperresponsiveness, and delayed adverse events could not be evaluated. Fourth, publication bias cannot be excluded, because most included studies were small Chinese-language trials, and funnel plot/Egger assessments may be underpowered or affected by heterogeneity. Fifth, the review was not prospectively registered, which may increase the risk of protocol-related bias. These limitations reduce the certainty and generalizability of the findings.

## 6. Conclusion

This meta-analysis shows that montelukast, when added to azithromycin sequential therapy, may improve overall clinical efficacy and accelerate symptom resolution in children with MPP without increasing short-term adverse events. Given the inflammatory nature of MPP and the rising challenge of macrolide resistance, integrating montelukast as an adjunct to sequential azithromycin therapy may represent a more comprehensive approach to managing pediatric MPP. However, the certainty of evidence was limited by methodological concerns, substantial heterogeneity in symptom-resolution outcomes, possible publication bias, and short follow-up durations. Further high-quality, multicenter RCTs with standardized treatment protocols and long-term follow-up are needed.

## Acknowledgments

The author wishes to express their gratitude to the team for their valuable contributions, help, and insightful discussions throughout the project.

## Author contributions

**Conceptualization:** Mingming Wu, Kan Chen, Haihong Yu.

**Data curation:** Mingming Wu, Kan Chen, Haihong Yu.

**Formal analysis:** Mingming Wu, Kan Chen, Haihong Yu.

**Funding acquisition:** Mingming Wu, Kan Chen, Haihong Yu.

**Investigation:** Mingming Wu, Haihong Yu.

**Writing – original draft:** Haihong Yu.

**Writing – review & editing:** Haihong Yu.
